# Ferroptosis-related genes in cervical cancer as biomarkers for predicting the prognosis of gynecological tumors

**DOI:** 10.3389/fmolb.2023.1188027

**Published:** 2023-04-28

**Authors:** Songtao Han, Senyu Wang, Xiang Lv, Dan Li, Yangchun Feng

**Affiliations:** ^1^ Xinjiang Key Laboratory of Oncology, Tumor Hospital Affiliated to Xinjiang Medical University, Ürümqi, China; ^2^ Clinical Laboratory Center, Hospital of Traditional Chinese Medicine Affiliated to Xinjiang Medical University, Ürümqi, China; ^3^ Department of Laboratory Medicine, Tumor Hospital Affiliated to Xinjiang Medical University, Ürümqi, China; ^4^ Department of Laboratory Medicine, Second Hospital Affiliated to Xinjiang Medical University, Ürümqi, China; ^5^ Department of Laboratory Medicine, Jianyang People’s Hospital, Chengdu, China; ^6^ Department of Encephalopathy, Hospital of Traditional Chinese Medicine Affiliated to Xinjiang Medical University, Ürümqi, China

**Keywords:** cervical cancer, ferroptosis, prognosis model, immune checkpoint, gynecological tumors

## Abstract

**Background:** Ferroptosis has been identified as a potent predictor of cancer prognosis. Currently, cervical cancer ranks among the most prevalent malignant tumors in women. Enhancing the prognosis for patients experiencing metastasis or recurrence is of critical importance. Consequently, investigating the potential of ferroptosis-related genes (FRGs) as prognostic biomarkers for cervical cancer patients is essential.

**Methods:** In this study, 52 FRGs were obtained from the GSE9750, GSE7410, GSE63514, and FerrDb databases. Six genes possessing prognostic characteristics were identified: JUN, TSC22D3, SLC11A2, DDIT4, DUOX1, and HELLS. The multivariate Cox regression analysis was employed to establish and validate the prognostic model, while simultaneously performing a correlation analysis of the immune microenvironment.

**Results:** The prediction model was validated using TCGA-CESC and GSE44001 datasets. Furthermore, the prognostic model was validated in endometrial cancer and ovarian serous cystadenocarcinoma cases. KM curves revealed significant differences in OS between high-risk and low-risk groups. ROC curves demonstrated the stability and accuracy of the prognostic model established in this study. Concurrently, the research identified a higher proportion of immune cells in patients within the low-risk group. Additionally, the expression of immune checkpoints (TIGIT, CTLA4, BTLA, CD27, and CD28) was elevated in the low-risk group. Ultimately, 4 FRGs in cervical cancer were corroborated through qRT-PCR.

**Conclusion:** The FRGs prognostic model for cervical cancer not only exhibits robust stability and accuracy in predicting the prognosis of cervical cancer patients but also demonstrates considerable prognostic value in other gynecological tumors.

## Introduction

Cervical cancer (CC) ranks as the fourth most common cancer among women worldwide (Sung et al., 2021). In 2020, approximately 600,000 new cases of this disease were reported, with around 340,000 deaths occurring globally (Sung et al., 2021). Persistent infection with human papillomavirus (HPV) can lead to precancerous cervical lesions, which may eventually progress to cancer (CrosbieEinsteinFranceschi and Kitchener, 2013). While the availability of HPV vaccines and cervical cancer screening has dramatically altered mortality and morbidity rates in high-income countries, coverage rates for these policies remain low in many low- and middle-income regions (only 10%) (Brüggmann et al., 2022; Bruni et al., 2022). The primary treatment for cervical cancer involves surgery or postoperative concurrent chemoradiotherapy. Metastasis or recurrence of the cancer substantially decreases the overall survival (OS) rate, which plummets to 5% at 4 years (Small et al., 2017). CC is considered one of the most lethal and threatening types of cancer among women globally, necessitating the development of novel tumor markers for accurate prognosis assessment.

Ferroptosis is an iron-dependent programmed cell death triggered by the accumulation of lipid-based reactive oxygen species (Dixon, 2017). Research has shown that the induction of ferroptosis can play various roles in signal transduction and bioregulation pathways, leading to tumor growth (Stockwell et al., 2017; Shen et al., 2018). Jiang et al. Xiaofei et al. (2021) discovered that the reduction in tumor size and decreased activity of Hela cells could be attributed to ACSL4-induced ferroptosis. Furthermore, FBXW7 (Zhang et al., 2020), G6PD (Dixon et al., 2012), and TP53 (Jiang et al., 2015a) promote ferroptosis in tumor cells, while CSD2 (Kim et al., 2018), GPX4 (Friedmann Angeli et al., 2014), and SLC7A11 (Jiang et al., 2015b) function as inhibitory factors to prevent ferroptosis. Wang et al. (2019) demonstrated that CD8^+^ T cells’ ability to enhance lipid peroxidation specific to ferroptosis could be harnessed for effective immunotherapies. The relationship between ferroptosis and immune cell infiltration holds potential for providing new insights into immunotherapeutic effectiveness.

Most current studies focus on bioinformatically analyzing the expression of ferroptosis-related genes or associated long non-coding RNAs (lncRNAs) in different cancer types to predict prognosis. Although ferroptosis-related genes (FRGs) have been identified as potential prognostic biomarkers for various cancer types, their evaluation in cervical cancer has not been conducted. The clinical information and expression data of patients were analyzed using the TCGA and GEO databases, with data from the FerrDb database also employed in the study. The aim of this study was to develop a prognostic model capable of evaluating the prognosis of women with cervical cancer and to test the model’s applicability to other gynecological tumors. Furthermore, the correlation between the prognostic model of FRGs and the immune microenvironment was analyzed, with FRGs validated by quantitative real-time PCR (qRT-PCR). This study’s objective was to establish a new strategy that could assist clinicians in predicting the prognosis of patients with cervical cancer.

## Materials and methods

### Sample and data collection

**FIGURE 1:**
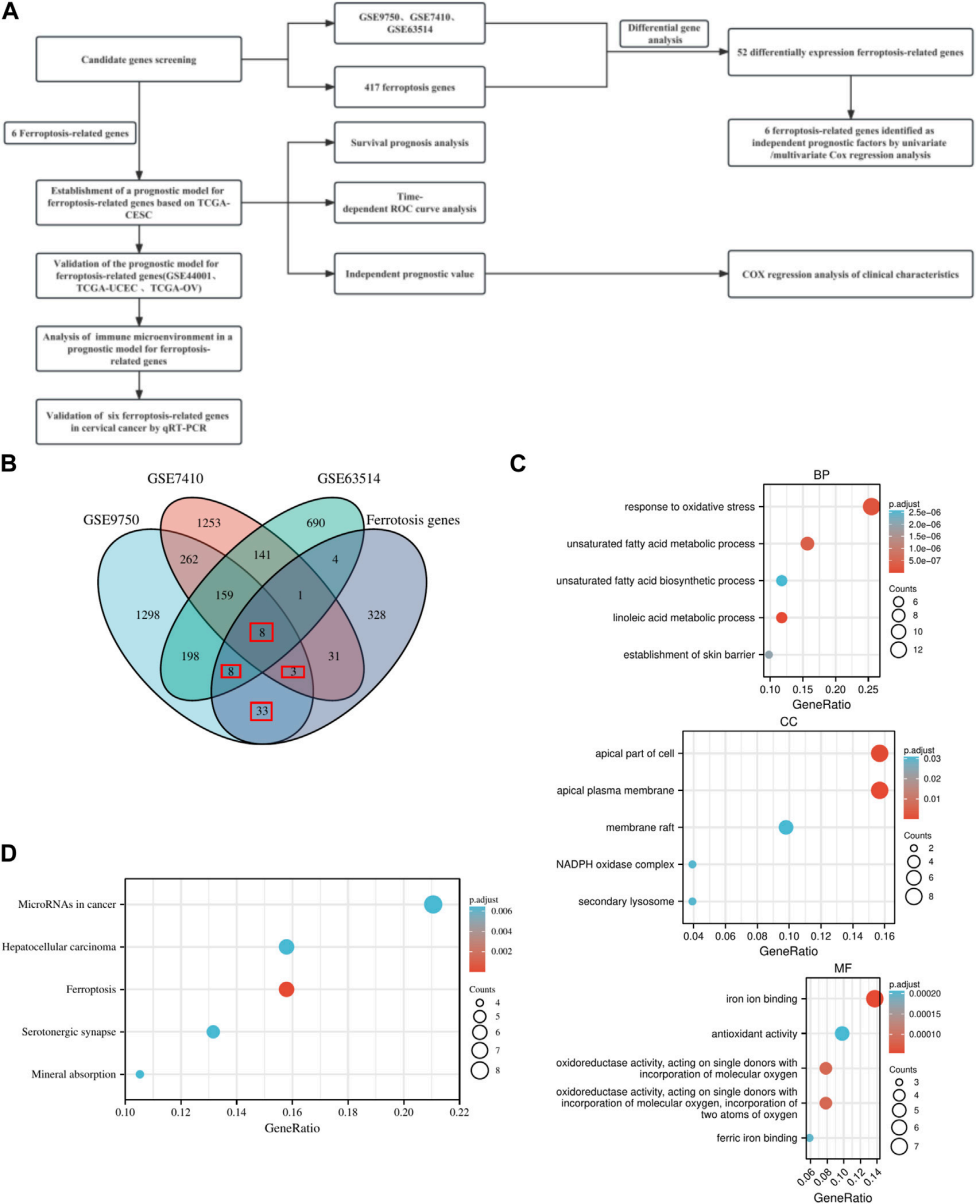
*Research plan and Overview of FRGs signatures*. **(A)** Research plan. **(B)** Venn diagram of differential genes and ferroptosis genes. **(C)** GO enrichment analysis of ferroptosis-related genes in BP, CC, and MF. **(D)** KEGG pathway enrichment analysis of ferroptosis-related genes.

The research plan is illustrated in Figure 1A. RNA transcriptome data and clinical information were acquired from the GEO and TCGA databases (http://www.ncbi.nlm.nih.gov/projects/geo/, https://portal.gdc.cancer.gov/). All data were transformed using log2 to ensure normalization. Four databases, FerrDb (http://www.zhounan.org/ferrdb/), NCBI-gene (https://www.ncbi.nlm.nih.gov/gene), MSigDB (http://www.gsea-msigdb.org/gsea/msigdb/), and Genecard (https://www.genecards.org/), provided a total of 416 ferroptosis genes (Supplementary Tables S1, S2).

### Differential expression and functional enrichment analysis

For the TCGA-CESC and GEO data, the R package edgeR conducted differential analysis on normal and cancer samples. The threshold was set at |Log (FC)|>1, p adj<0.05, and the intersection of differentially expressed genes and ferroptosis genes was determined (The criteria for selecting overlapping genes required their presence in at least two datasets, with one dataset being a ferroptosis gene set). The R package clusterProfiler (version 3.14.3) performed GO/KEGG enrichment analysis of differentially expressed FRGs.

### Prognostic model Establishment and prognostic analysis

The R package (glmnet version 4.1.1) executed LASSO regression on the differentially expressed FRGs to filter out redundant factors. Subsequently, univariate/multivariate Cox regression analysis determined the prognostic genes and constructed a Risk score prognostic model (The majority of literature calculates Risk score based on the weighting of the product of gene expression and its coefficients. This study employed multivariate Cox regression to develop a model in which Risk score was determined as the weighting of the product of gene expression and its coefficients.). High-risk groups (n = 153) and low-risk groups (n = 153) were categorized according to the median of the Risk score. The R packages survival ROC (version 1.0.3) and rms (version 6.2.0) analyzed 1-year, 3-year, 5-year survival prognoses and prognostic risk performance.

$$riskscore=\sum_{i=1}^{n}\beta i\times \mathrm{Exp}\left(i\right)$$

### Clinicopathological features and immune infiltration analysis

The correlation between the Risk score, constructed by FRGs, and clinicopathological characteristics was assessed. Immune infiltration analysis was performed by ssGSEA algorithm to obtain enrichment scores for each class of immune cells in each sample of TCGA-CESC and GSE44001. The Risk score was then divided into high and low risk groups based on the median of the Risk score among all samples. Differences in the enrichment scores of 24 immune cells (Gabriela et al., 2013) in high and low risk groups were assessed to infer the composition of immune cells in patients with cervical cancer under Risk score.

### qRT-PCR detection

A total of 25 cervical cancer tissue samples were collected from surgical patients at the Tumor hospital affiliated with Xinjiang Medical University between 2015 and 2020, with signed informed consent forms. The study was approved by the Ethics Committee of the Tumor hospital affiliated with Xinjiang Medical University and conformed to the Helsinki Declaration and Clinical Practice Guidelines. Total RNA extraction from tissues was performed using TRIzol reagent (Invitrogen, United States), and cDNA synthesis occurred by reverse transcription using the PrimeScript real-time kit (Takara, Japan). qRT-PCRs were conducted using an ABI 7500 PRISM 7500 Platform (Applied Biosystems, United States). GAPDH served as a reference, and relative expression levels of target genes were calculated employing the 2^−ΔΔCt^ method. Primers for correlation analysis can be found in Supplementary Tables S3.

### Statistical analysis

Differential analysis of normal and cancer samples was conducted using the R package edgeR, with a threshold of |Log (FC)|>1 and p adj<0.05. The R package implemented LASSO regression and univariate/multivariate Cox regression analysis on differentially expressed FRGs. The R packages survminer (version 0.4.9) and survival ROC (version 1.0.3) performed KM(Cox regression was used for analysis) and ROC curve analysis to predict the survival prognosis of patients with cervical cancer. Correlation analysis of survival Risk scores constructed from FRGs and clinicopathological characteristics utilized univariate and multivariate Cox regression analysis. Differences in genes were analyzed by independent samples t-test and visualized using GraphPad Prism 8. The test level was α = 0.05, and a difference was considered statistically significant with *p* < 0.05.

## Results

### Screening and functional analysis of FRGs

Differential gene analysis yielded 1,969, 2,142, and 1,209 differential genes for GSE9750, GSE7410, and GSE63514, respectively (Supplementary Figure S1; Supplementary Table S2). In three distinct datasets, the DEGs were combined with ferroptosis genes to generate differentially expressed FRGs. A total of 52 FRGs were obtained (Figure 1B; Supplementary Tables S4). Ultimately, 52 FRGs underwent GO/KEGG enrichment analysis (Supplementary Tables S5). Enrichment analysis in biological process (BP), cellular component (CC), and molecular function (MF) domains indicated that the gene set was involved in various activities, including the apical part of the cell, iron ion binding, and response to oxidative stress (Figure 1C). KEGG pathway enrichment analysis revealed significant enrichment of the gene set in both ferroptosis and cancer-related pathways (Figure 1D).

### Establishment and prognostic analysis of FRGs prognostic model in cervical cancer

**FIGURE 2:**
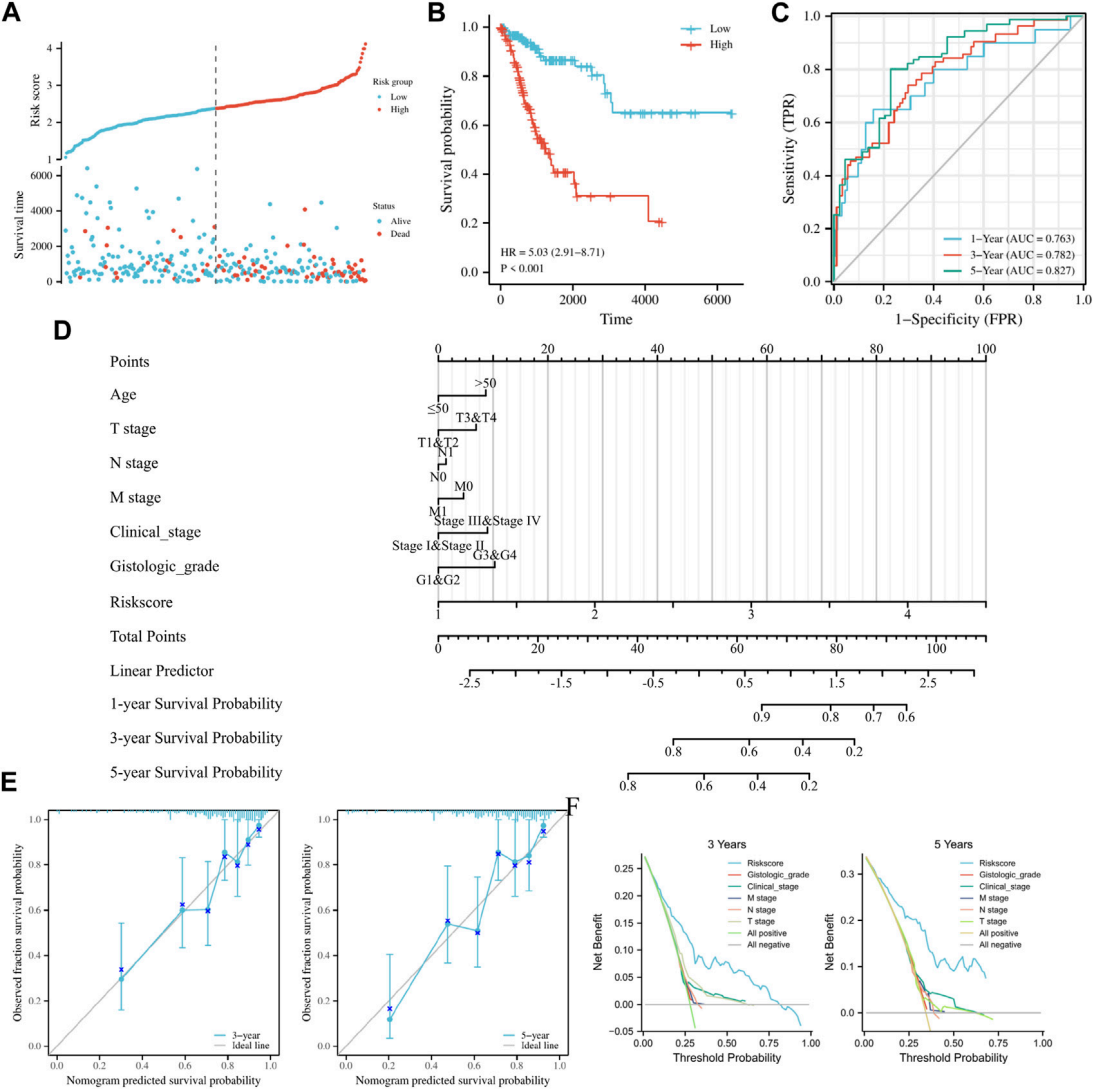
*Establishment and prognostic analysis of FRGs prognostic model in cervical cancer*. **(A)** Curve scatter plot of training set survival model efficacy assessment and cumulative scatter plot of survival mortality event risk. **(B)** KM curves show a significant difference in OS between high-risk and low-risk groups in the training set. **(C)** Time-dependent ROC curves were used to predict 1, 3 and 5 years survival. **(D)** 1, 3, and 5 years nomograms for predicting OS in cervical cancer. **(E)** Calibration curves showing the agreement between predicted and observed 3 and 5 years OS. **(F)** Decision curve analysis of the prognostic model in the training set at 3 and 5 years.

TCGA-CESC data served as the training set. Initially, 15 FRGs were derived from LASSO analysis of the 52 FRGs (Supplementary Figure S2). Subsequently, Cox regression analysis results demonstrated that among eight FRGs, six exhibited independent effects on predicted outcomes, including JUN, TSC22D3, SLC11A2, DDIT4, DUOX1, and HELLS (Table 1). A prognostic model was developed based on these six genes and classified into two groups according to Risk score. Scatter plots of survival outcomes and survival time indicated that the high-risk group had more fatalities than the low-risk group (Figure 2A). The training set’s KM curve showed that the OS of the low-risk group was longer than that of the high-risk group (*p* < 0.001, Figure 2B). ROC curves were employed to analyze the OS at 1, 3, and 5 years, with AUC values of 0.763, 0.782, and 0.827, respectively (Figure 2C). In conclusion, the model provided a stable and accurate prediction of patients' prognosis.

**TABLE 1:** Univariate/multivariate Cox regression analysis of FRGs.

Characteristics	Total(N)	Univariate analysis		Multivariate analysis	
		Hazard ratio (95% CI)	*p*-value	Hazard ratio (95% CI)	*p*-value
*JUN*	306	1.873 (1.167–3.005)	**0.009**	1.879 (1.154–3.059)	**0.011**
DNAJB6	306	1.710 (1.070–2.735)	**0.025**	1.581 (0.973–2.569)	0.064
*TSC22D3*	306	0.605 (0.380–0.965)	**0.035**	0.457 (0.277–0.754)	**0.002**
*SLC11A2*	306	1.611 (1.004–2.585)	**0.048**	1.855 (1.129–3.048)	**0.015**
*DDIT4*	306	2.097 (1.294–3.398)	**0.003**	2.416 (1.413–4.130)	**0.001**
*DUOX1*	306	0.439 (0.270–0.712)	**<0.001**	0.454 (0.274–0.754)	**0.002**
CA9	306	1.644 (1.029–2.628)	**0.038**	1.128 (0.685–1.856)	0.635
*HELLS*	306	0.529 (0.329–0.851)	**0.009**	0.488 (0.288–0.828)	**0.008**

### Prognostic analysis of clinicopathological features by FRGs prognostic model in cervical cancer

Clinicopathological features of the study were examined using univariate/multivariate Cox regression analyses. According to univariate analysis, clinical stage, TNM stage, and Risk score were significant factors predicting patient prognosis. In contrast, multivariate analysis revealed that Risk score was the sole independent factor predicting patient prognosis (Table 2). To ensure accurate patient prognosis, a nomogram incorporating various clinicopathologic parameters was generated (Figure 2D). Additionally, DCA and calibration curves (Figures 2E, F) demonstrated the model’s role in assessing patient outcomes. In summary, the model could be employed as a novel and powerful tool for predicting patient prognosis.

**TABLE 2:** Univariate/multivariate Cox regression analysis of clinicopathological characteristics of TCGA-CESC.

Characteristics	Total (N)	Univariate analysis		Multivariate analysis	
		Hazard ratio (95% CI)	*p*-value	Hazard ratio (95% CI)	*p*-value
Age(>50 *vs*. ≤ 50)	306	1.289 (0.810–2.050)	0.284	0.577 (0.157–2.122)	0.408
T stage (T3&T4 *vs*. T1&T2)	243	3.863 (2.072–7.201)	**<0.001**	2.148 (0.325–14.219)	0.428
N stage(N1 *vs*. N0)	195	2.844 (1.446–5.593)	**0.002**	1.223 (0.352–4.248)	0.751
M stage(M1 *vs*. M0)	127	3.555 (1.187–10.641)	**0.023**	0.000 (0.000-Inf)	0.998
Clinical stage (Stage III&Stage IV *vs*. Stage I&Stage II)	299	2.369 (1.457–3.854)	**<0.001**	1.282 (0.133–12.361)	0.830
Histologic grade (G3&G4 *vs*. G1&G2)	274	0.866 (0.514–1.459)	0.589	2.396 (0.663–8.654)	0.182
RiskScore (High *vs*. Low)	294	8.191 (1.081–62.076)	**0.042**	14.075 (1.147–172.783)	**0.039**

### Validation and prognostic efficacy analysis of prognostic model of FRGs in cervical cancer

**FIGURE 3:**
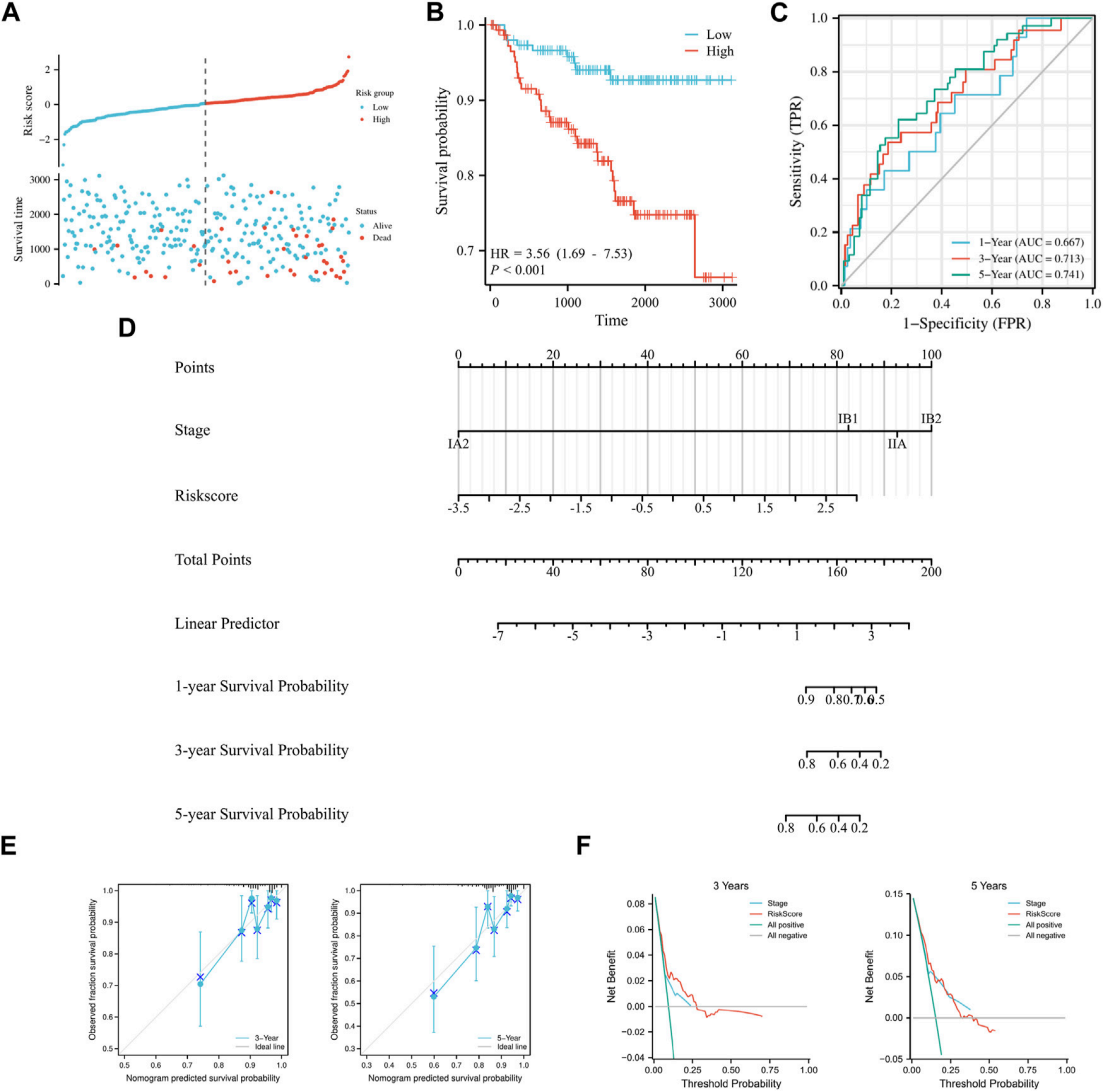
*Validation of a prognostic model of FRGs in cervical cancer.* **(A)** Curve scatter plot of validation set survival model efficacy assessment and cumulative scatter plot of survival mortality event risk. **(B)** KM curve shows that there is a significant difference in OS between high-risk and low-risk groups in GSE44001. **(C)** Time-dependent ROC curves were used to predict 1, 3, and 5 years survival. **(D)** 1, 3, and 5 years nomograms for predicting OS in cervical cancer. **(E)** Calibration curves showing the agreement between predicted and observed 3 and 5 years OS. **(F)** Decision curve analysis of the prognostic model in the validation set at 3 and 5 years.

To validate the model’s applicability, the GSE44001 dataset was used as the validation set. A prognostic model was developed based on the six aforementioned genes, which were divided into high and low groups according to median Risk score. Scatter plots of survival outcomes and survival time indicated that the high-risk group had more fatalities than the low-risk group (Figure 3A). The validation set’s KM curve showed that the OS of the low-risk group was longer than that of the high-risk group (*p* < 0.001, Figure 3B). ROC curves were employed to analyze the OS at 1, 3, and 5 years, with AUC values of 0.667, 0.713, and 0.741, respectively (Figure 3C). The study results also indicated that the model was stable and accurate in the validation set. Univariate/multivariate Cox regression analyses were then employed with the dataset to further validate the model’s clinicopathologic characteristics. According to univariate analysis results, Risk score and IB2 were the primary prognostic factors. In multivariate regression analysis, Risk score was considered an independent predictor of the study’s outcome (Table 3). A nomogram was also used to evaluate the model’s value in assessing patient prognosis in the GSE44001 dataset (Figure 3D). DCA and calibration curves (Figures 3E, F) also demonstrated that the model had a consistent effect on patient prognosis assessment. In conclusion, the model’s practicality and suitability for various datasets render it an ideal choice for determining cervical cancer patients' prognosis.

**TABLE 3:** Univariate/multivariate Cox regression analysis of clinicopathological characteristics of GSE44001.

Characteristics	Total(N)	Univariate analysis		Multivariate analysis	
		Hazard ratio (95% CI)	*p*-value	Hazard ratio (95% CI)	*p*-value
Stage	300				
IB1	217	Reference			
IA2	13	0.000 (0.000-Inf)	0.996	0.000 (0.000-Inf)	0.996
IB2	28	3.953 (1.807–8.651)	**<0.001**	3.038 (1.334–6.920)	**0.008**
IIA	42	2.106 (0.932–4.758)	0.073	1.920 (0.844–4.369)	0.120
RiskScore (High *vs*. Low)	300	2.718 (1.810–4.081)	**<0.001**	2.270 (1.539–3.349)	**<0.001**

### Validation and prognostic efficacy analysis of FRGs prognostic model for cervical cancer in other gynecological tumors

**FIGURE 4:**
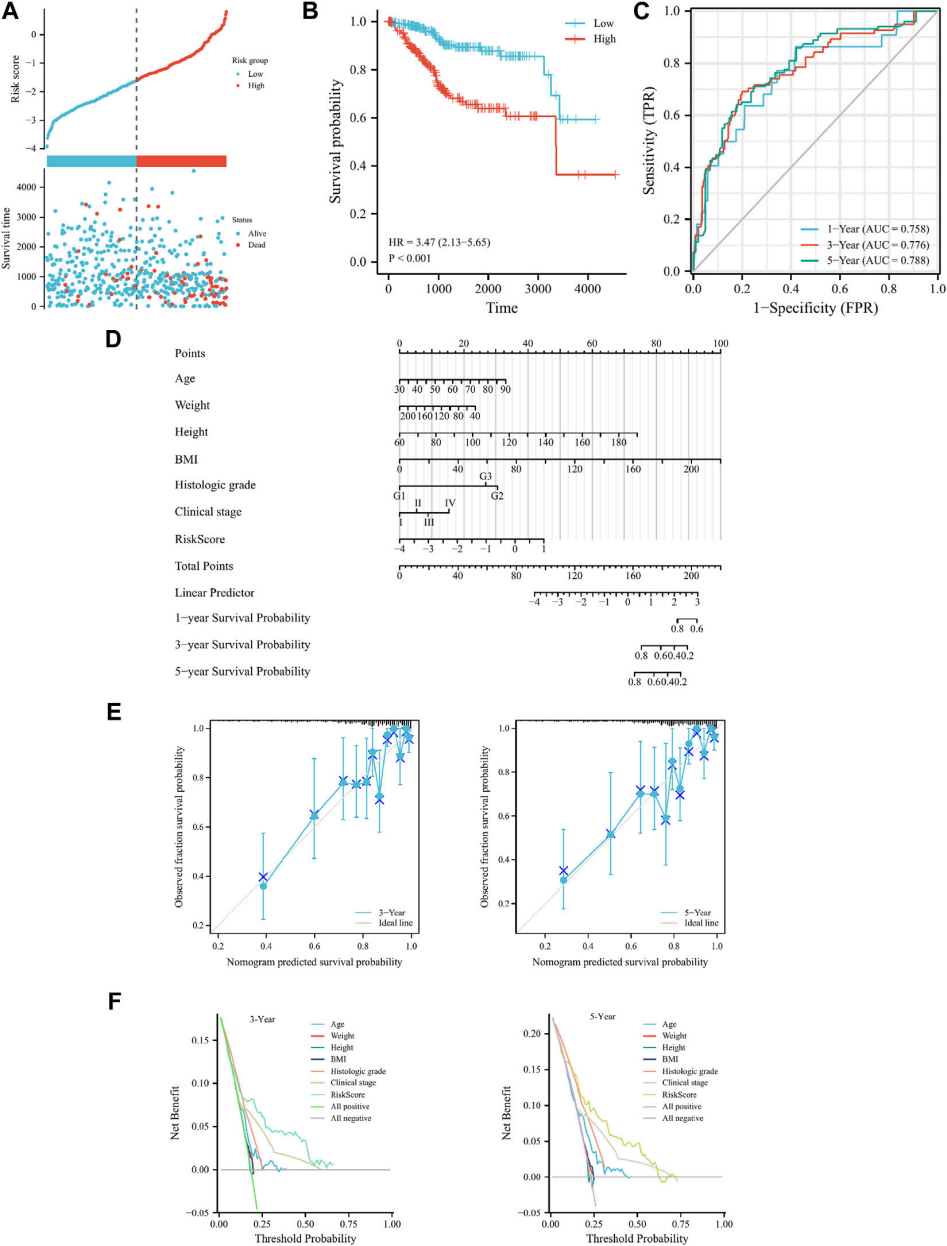
*Cervical cancer FRGs prognosis model validation in the TCGA-UCEC.* **(A)** Curve scatter plot of validation set survival model efficacy assessment and cumulative scatter plot of survival mortality event risk. **(B)** KM curve shows that there is a significant difference in OS between high-risk and low-risk groups in TCGA-UCEC. **(C)** Time-dependent ROC curves were used to predict 1, 3, and 5 years survival. **(D)** 1, 3 and 5 years nomograms for predicting OS in TCGA-UCEC. **(E)** Calibration curves showing the agreement between predicted and observed 3 and 5 years OS. **(F)** Decision curve analysis of the prognostic model in the validation set at 3 and 5 years.

To confirm the model’s universal applicability across different gynecological tumors, TCGA-UCEC and TCGA-OV datasets were employed as validation sets. Scatter plots of survival outcomes and survival time indicated that the high-risk group had more fatalities than the low-risk group (Figure 4A). The validation set’s KM curve revealed that the OS of the low-risk group was longer than that of the high-risk group in TCGA-UCEC (*p* < 0.001, Figure 4B). ROC curves were utilized to analyze the OS at 1, 3, and 5 years, with AUC values of 0.758, 0.776, and 0.788, respectively (Figure 4C). The results demonstrated that the model exhibited excellent stability and accuracy in TCGA-UCEC. Clinicopathological features of the model were further verified using univariate/multivariate Cox regression analysis on the TCGA-UCEC dataset. Univariate/multivariate regression analysis revealed that Age, Clinical stage, Histologic grade, and risk score were prognostic factors for TCGA-UCEC (Table 4). Ultimately, a nomogram was established to predict the OS of cervical cancer patients in the TCGA-UCEC dataset at 1, 3, and 5 years (Figure 4D). The DCA diagram and calibration curve (Figures 4E, F) also confirmed that the nomogram combined with clinical features held significant clinical application value. Additionally, the same analysis was performed on the TCGA-OV dataset, which indicated that the cervical cancer prognostic model of iron death-related genes also possessed robust prognostic value in ovarian serous cystadenocarcinoma (Supplementary Figure S2; Supplementary Table S6). In conclusion, the cervical cancer prognostic model of iron death-related genes exhibits strong applicability and can serve as biomarkers to predict patient prognosis across different gynecological tumor datasets.

**TABLE 4:** Univariate and multivariate Cox regression analysis of clinicopathological characteristics of TCGA-UCEC.

Characteristics	Total (N)	Univariate analysis		Multivariate analysis	
		Hazard ratio (95% CI)	*p*-value	Hazard ratio (95% CI)	*p*-value
Age(>60 *vs*. <=60)	549	0.541 (0.340–0.862)	**0.010**	0.484 (0.293–0.799)	**0.005**
Weight(>80 *vs*. <=80)	527	0.944 (0.622–1.431)	0.784	0.519 (0.228–1.185)	0.119
Height(>160 *vs*. <=160)	522	0.868 (0.571–1.319)	0.507	0.907 (0.567–1.450)	0.682
BMI(>30 *vs*. <=30)	518	1.034 (0.680–1.572)	0.876	1.479 (0.674–3.243)	0.329
Clinical stage (Stage III&Stage IV *vs*. Stage I&Stage II)	551	0.282 (0.188–0.425)	**<0.001**	0.269 (0.171–0.424)	**<0.001**
Histologic grade (G3 *vs*. G1&G2)	540	0.305 (0.177–0.524)	**<0.001**	0.409 (0.232–0.719)	**0.002**
RiskScore (High *vs*. Low)	506	2.718 (2.124–3.479)	**<0.001**	2.060 (1.196–3.548)	**0.009**

### Correlation analysis between prognostic model of FRGs and immune microenvironment

**FIGURE 5:**
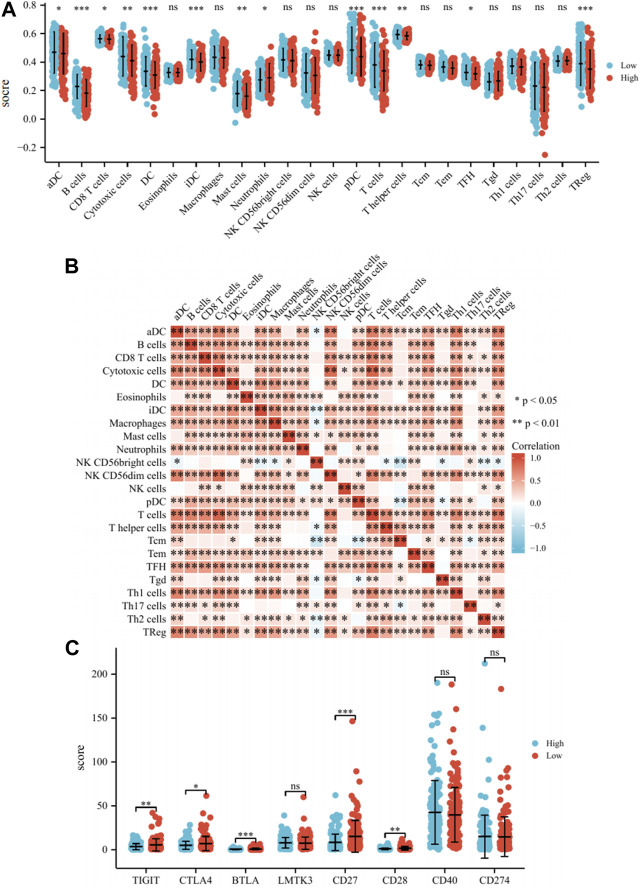
*Analysis of the immune microenvironment.* **(A)** Differences of 24 immune cells in different expression levels of Risk score. **(B)** Correlation between 24 immune cells. **(C)** Differences of immune checkpoint inhibitors in different expression levels of Risk score. Note: ns: no significant difference, *: *p* < 0.05, **: *p* < 0.01, ***: *p* < 0.001.

We found that the low-risk group had significantly higher enrichment scores for B cells, DC, iDC, pDC, T cells, and TReg than the high-risk group (*p* < 0.001, Figure 5A). The enrichment scores in Cytotoxic cells, Mast cells, and T helper cells were slightly higher than the high-risk group (*p* < 0.01, Figure 5A). The enrichment scores for aDC, CD8+T cells, Neutrophils, and TFH were not significantly higher than the high-risk group (*p* < 0.05, Figure 5A). It can be seen that the immune microenvironment of cervical cancer patients under Risk score consists of immune cells such as B cells, T cells, DC and mast cells. Lastly, the correlations between 24 immune cells were assessed, and the correlations between different tumor-infiltrating immune cell subsets ranged from weak to moderate correlations (Figure 5B). The same analysis was conducted for GSE44001, with statistically significant enrichment scores for Macrophages, Mast cells, and Neutrophils, which also resembled the TCGA cohort (Supplementary Figure S4). The expression of various immune checkpoint inhibitors, such as CTLA4, BTLA, CD27, CD28, and CD40, in high and low-risk groups was also analyzed. The results demonstrated that the expression of TIGIT, CTLA4, BTLA, CD27, and CD28 were higher in the low-risk group than in the high-risk group, indicating improved immune efficacy for patients in the low-risk group. The level of expression of other checkpoint inhibitors was not significantly different between the two groups (Figure 5C). In conclusion, the model correlates with the prognosis of patients with cervical cancer from an immune infiltration perspective. Simultaneously, the high expression of immune checkpoint inhibitors in the low-risk group enhances the effectiveness of immunotherapy in patients.

### Validation of FRGs expression levels

**FIGURE 6:**
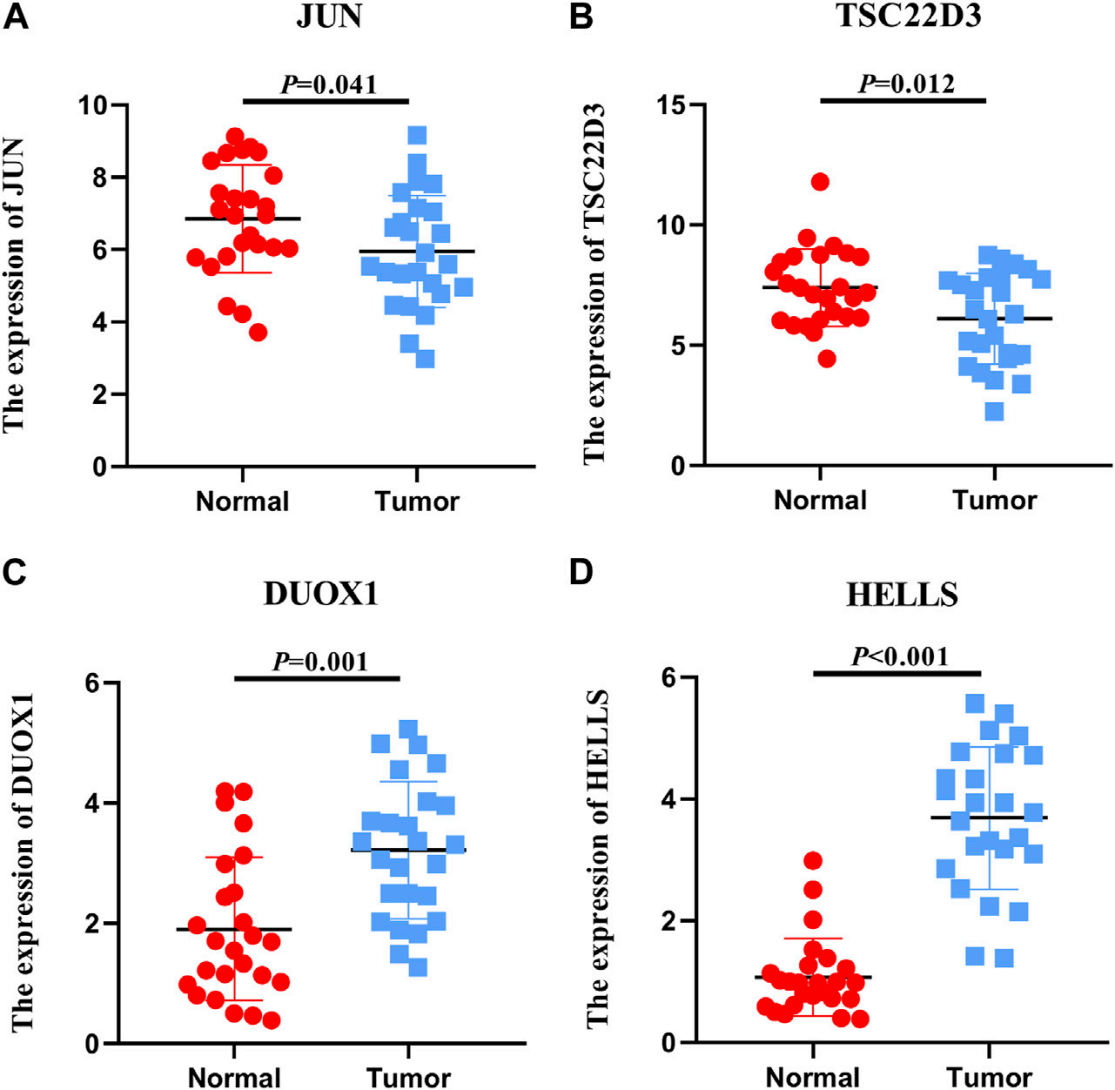
*Expression levels of FRGs*. Expression levels of 4 ferroptosis-related genes in 25 non-tumor tissues and 25 cervical cancer tissues.

The expression levels of the model’s six genes were validated using the TCGA database. The expression levels of DDIT4 and SLC11A2 were not significantly different when comparing noncancerous and cancerous tissues (Supplementary Figure S5). qRT-PCR analysis was performed to assess the levels of the remaining genes in both cervical and non-cancerous tissues. Consequently, the expression levels of JUN and TSC22D3 in cervical cancer tissues displayed an overall downward trend compared with non-tumor tissues (Figures 6A, B). DUOX1 and HELLS exhibited an overall upward trend (Figures 6C, D). Furthermore, the relationship between four genes and B cells, CD8 T cells, DC, NK cells, and T cells was examined, revealing that TSC22D3 was positively correlated with the aforementioned cells; aside from NK cells, HELLS was negatively correlated with the other 4 cells; DUOX1 was negatively correlated with CD8+T cells and NK cells but positively correlated with DC; JUN was negatively correlated with B cells and showed no correlation with other cells (Supplementary Figure S6). In summary, the four genes exhibit specific expression in cervical cancer tissue, and there is a discernible correlation with immune infiltration.

## Discussion

Despite the progress made in the prevention, screening, and treatment of cervical cancer, the outcomes of the disease have not significantly improved (Zeng et al., 2018). For cervical cancer patients with metastasis or recurrence, the 5-year OS is only 17% (Ouyang et al., 2020). Currently, the main research focus in ferroptosis is on the occurrence, development, and treatment of tumors. Several studies have demonstrated that ferroptosis-related biomarkers are strong predictors of cancer prognosis and treatment efficacy (Shi et al., 2019; Liang et al., 2020; Tang et al., 2020). Based on these findings, it is essential to systematically and comprehensively evaluate the prognostic role of FRGs in cervical cancer.

In this study, the impact of FRGs prognostic models on prognosis was investigated, while also examining the relationship between FRGs prognostic models and the immune microenvironment to determine if this model could be a potential biomarker for prognosis. Initially, the DEGs of the GSE9750, GSE7410, and GSE63514 datasets were analyzed. The intersection of the ferroptosis gene sets was obtained from the FerrDb, NCBI-gene, MSigDB, and Genecard databases. Notably, as the intersection of the four datasets yielded fewer genes, the intersection of each dataset was analyzed, resulting in a total of 52 FRGs. Then, functional analysis of these 52 FRGs showed that they were related to ferroptosis and oxidative stress processes. Univariate/multivariate Cox regression analysis was employed to identify FRGs with prognostic features and to establish a prognostic model for FRGs. Subsequently, the ssGSEA was used to study the differences between various immune cells. Statistically significant differences were found for B cells, DC, iDC, pDC, T cells, and TReg.

Ferroptosis is currently recognized as an immunogenic cell death characterized by the release of damage-associated molecular patterns (DAMPs) from deceased tumor cells (Tang et al., 2019; Wen et al., 2019; Wan et al., 2020). The analysis discovered that B cells, DC, T cells, and TReg exhibited higher abundances in the low-risk group compared to the high-risk group, which displayed a higher immune score. These correlation results demonstrate, to some extent, the relevance of FRGs prognostic models to the immune infiltration of cervical cancer. This can be combined with the finding by Wang et al. (2019) that the antitumor efficacy of immunotherapy can be achieved through enhanced ferroptosis-specific lipid peroxidation by CD8^+^ T cells. Additionally, the analysis of immune checkpoint inhibitors revealed higher expression of TIGIT, CTLA4, BTLA, CD27, and CD28 in the low-risk group, suggesting that the efficacy of immunotherapy is better in the low-risk group than in the high-risk group. TIGIT, an emerging immune checkpoint, is widely expressed on lymphocytes (Harjunpää and Guillerey, 2020). It is capable of inhibiting every step of the cancer immune cycle (Manieri et al., 2017). TIGIT may prevent NK cells from releasing tumor antigens, impair DC-primed T cell priming, or inhibit CD8+T cell-mediated cancer cell killing (Harjunpää and Guillerey, 2020). Combined with the results of this study, it is plausible that TIGIT kills cancer cells in the low-risk group by reducing DC-triggered T-cell initiation, leading to immunotherapeutic benefits for patients in the low-risk group. However, further research is needed to elucidate the specific mechanism.

In this study, four FRGs, including JUN, TSC22D3, DUOX1, and HELLS, were experimentally validated and analyzed for correlation with immune cells. TSC22D3 is a transcriptional regulator that mediates immunosuppressive effects through NF-κB, RAS, and other pathway proteins, as well as heterodimerization ability (Ronchetti et al., 2015). It has been shown that elevated glucocorticoids due to stress induce the expression of TSC22D3, which blocks type I interferon (IFN) responses and IFN-γ+ T cell activation in dendritic cells (DCs), thereby disrupting immune surveillance (Yang et al., 2019a). Based on previous findings, and considering the positive correlation of TSC22D3 with immune cells in this study, it is reasonable to suspect that in cervical cancer, TSC22D3 expression may enhance the immunity of patients by stimulating the activation of immune cells, thereby prolonging their prognosis. DUOX1 is expressed at low levels in HCC and can be used as an important indicator for evaluating the therapeutic effect of HCC after surgery (Lu et al., 2011). However, DUOX1 is overexpressed in patients with cervical cancers (Cho et al., 2019). DUOX1 was strongly correlated with the ratios of CD8^+^ T cells, DCs, and NK cells, indicating that its expression was highly associated with the innate immune cell response in cervical cancer. Furthermore, DUOX1 expression in innate lymphocytes suggests that DUOX1 has a broad host defense function (Habibovic et al., 2016; Cho et al., 2019), resulting in prolonged survival prognosis for patients with cervical cancer. HELLS is overexpressed in colorectal, HCC, nasopharyngeal, and lung cancers, leading to poorer prognosis, and therefore, HELLS can be useful as a prognostic marker in various cancers (He et al., 2016; Yang et al., 2019b; Law et al., 2019; Liu et al., 2019; Zhu et al., 2020; Xing et al., 2021). Zocchi et al. (2020) found that low expression of HELLS in retinoblastoma inhibited ectopic division of differentiated cells in the retina, leading to tumor development inhibition and, consequently, prolonging OS in patients (Zocchi et al., 2020; Xing et al., 2021). In conjunction with previous studies, HELLS displayed a negative correlation with B cells, CD8 T cells, DC, and T cells in this investigation, with high HELLS expression signifying reduced expression of immune cells and promotion of tumor progression. As a critical prognostic gene in this study, it is valuable to delve deeper into how patients’ prognosis can be enhanced through the mechanism of HELLS.

In addition to this study, it is noteworthy that Du et al. (2022), Qin et al. (2022), Qi et al. (2021), and Xing et al. (2021) all investigated FRGs in cervical cancer. Du et al. (2022) constructed a prognostic model with excellent predictive performance based on FRGs. CA9/ULBP2 was also identified as a potential regulator of cervical carcinogenesis and progression. Qin et al. (2022) constructed a prognostic model with four iron death-associated genes and examined the immune microenvironment. Qi et al. (2021) developed a novel prognostic model with FRGs and validated the genes within the model. Xing et al. (2021) constructed a model with immune-associated genes and iron death genes related to OS in CESC patients, effectively predicting the outcome.

It is worth mentioning that most of the above studies selected 1-2 datasets for analysis and model construction. In this study, FRGs were obtained from multiple datasets, and a model was built. The model demonstrates good stability and accuracy in TCGA-CESC and GSE44001 datasets. Furthermore, it has significant predictive value and general applicability in other gynecological tumors. In addition, the expression of four genes, including JUN, TSC22D3, DUOX1, and HELLS, in cervical cancer tissues was verified by qRT-PCR. However, considering the limitations of previous related research, further study is necessary to explore the immune molecular mechanism between ferroptosis and cervical cancer and how this mechanism affects the prognosis of patients with cervical cancer.

Of course, this study had two limitations. First, one of the cohorts included relatively few indicators in clinical information, leading to insufficient validation of some results. Second, the study used retrospective data from public databases to construct and validate a prognostic model for FRGs. It would be more convincing to use prospective data to assess its clinical utility. Based on these two points, combined with the current lack of understanding of the mechanism of genes in cervical cancer, it is essential to further explore and study the biological functions of genes in cervical cancer in future research.

In conclusion, this study fills the gap in the FRGs prognostic model for cervical cancer prognosis. The constructed prognostic model possesses a strong ability to predict the survival outcome of patients with cervical cancer and has certain applicability to other gynecological tumors. Ultimately, the model demonstrates a correlation with the prognosis of cervical cancer patients in terms of immune infiltration, and the high expression of immune checkpoint inhibitors in the low-risk group is more conducive to immunotherapy efficacy. It is hoped that these findings will provide new insights for future research and clinical practice.

## Conclusion

In this research, FRGs were derived from multiple datasets, and a cervical cancer prognostic model was developed. This model was validated not only in external cervical cancer datasets but also in datasets of other gynecological tumors. Simultaneously, 4 FRGs were confirmed using qRT-PCR. The association between immune infiltration and patient prognosis, as well as the differences in the expression of immune checkpoint inhibitors under varying risk scores, were also determined in this study.

## Data Availability

The original contributions presented in the study are included in the article/Supplementary Material, further inquiries can be directed to the corresponding author.

## Supplementary material

SUPPLEMENTARY FIGURE S1 Volcano plot display for GSE9750, GSE7410, GSE63514 datasets.

SUPPLEMENTARY FIGURE S2 Lasso Cox analysis (A: Lasso coefficient B: Lasso variable track).

SUPPLEMENTARY FIGURE S3 Validation of FRGs in cervical cancer in TCGA-OV (A: Curve scatter plot of training set survival model efficacy assessment and cumulative scatter plot of survival mortality event risk. B: KM curves show a significant difference in OS between high-risk and low-risk groups in TCGA-OV. C: Time-dependent ROC curves were used to predict 1, 3, and 5 years survival. D: 1, 3, and 5 years nomograms for predicting OS in cervical cancer. E: Calibration curves showing the agreement between predicted and observed 3 and 5 years OS. F: Decision curve analysis of the prognostic model in the training set at 3 and 5 years.).

SUPPLEMENTARY FIGURE S4 GSE44001 immune infiltration analysis (A: Differences of 24 immune cells in different expression levels of Risk score. B: Correlation between 24 immune cells.).

SUPPLEMENTARY FIGURE S5 Six genes were analyzed in TCGA database (A: the expression of JUN B:the expression of TSC22D3 C: the expression of SLC11A2 D: the expression of DDIT4 E: the expression of DUOX1 F: the expression of HELLS).

SUPPLEMENTARY FIGURE S6 Analysis of immune infiltration of 4 genes (A: Correlation between JUN and immune cells B: Correlation between TSC22D3 and immune cells C: Correlation between DUOX1 and immune cells D: Correlation between HELLS and immune cells).

SUPPLEMENTARY TABLE S1 The information of datasets from the GEO database.

SUPPLEMENTARY TABLE S2 GSE9750, GSE7410, GSE63514 and Ferroptosis genes dataset differential genes display.

SUPPLEMENTARY TABLE S3 qRT-PCR primers for mRNA or miRNA expression analysis.

SUPPLEMENTARY TABLE S4 Wayne chart genetics showcase.

SUPPLEMENTARY TABLE S5 GO/KEGG enrichment analysis demonstration.

SUPPLEMENTARY TABLE S6 Univariate and multivariate Cox regression analysis of clinicopathological characteristics of TCGA-OV.
