# Supplementary figures and images for: Ferroptosis-related genes in cervical cancer as biomarkers for predicting the prognosis of gynecological tumors
Source: Front Mol Biosci. 2023 Apr 28;10:1188027. doi: 10.3389/fmolb.2023.1188027 (PMC10175786; doi:10.3389/fmolb.2023.1188027)

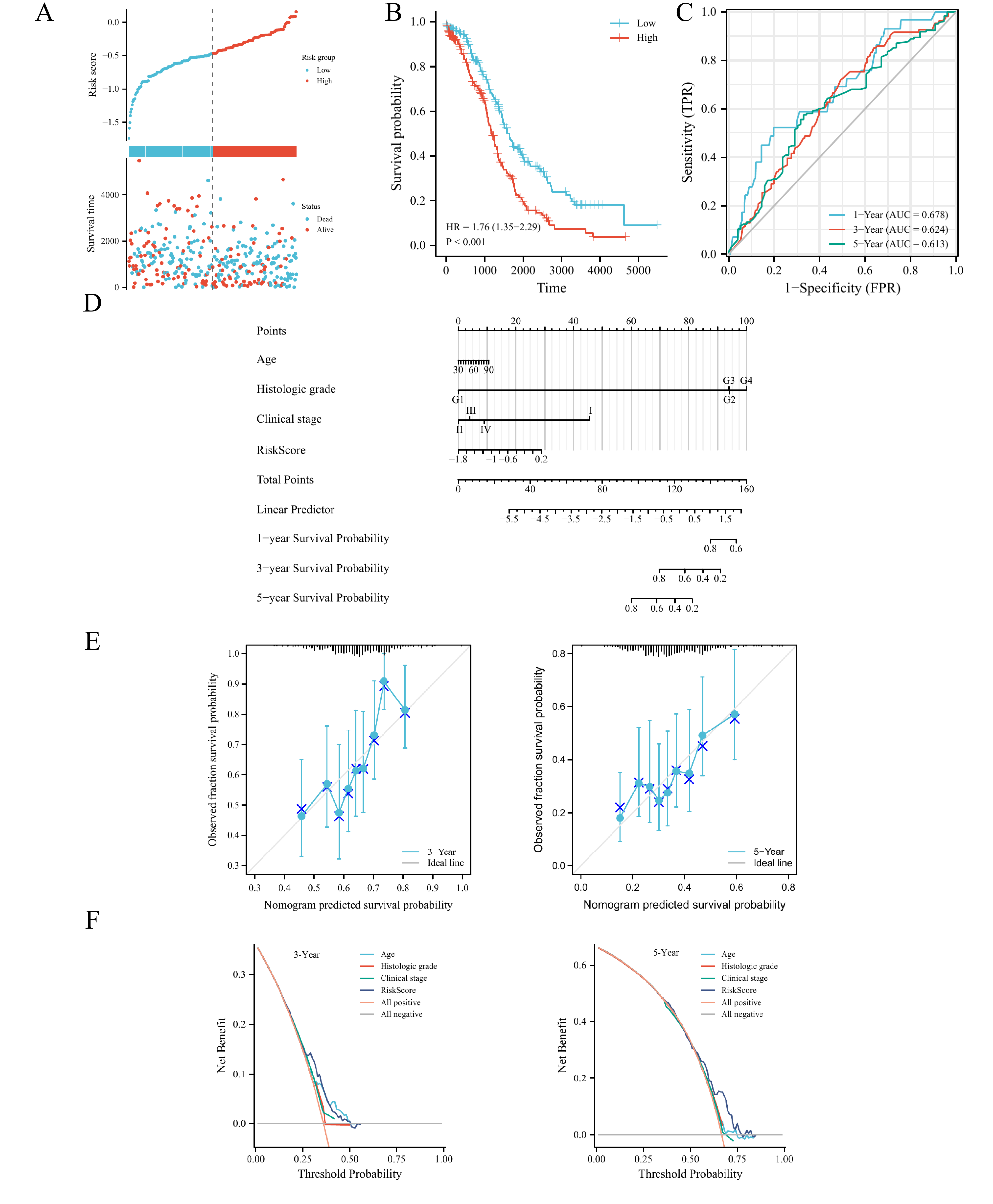

Supplement: Supplementary file 1 [file Image3.TIFF]

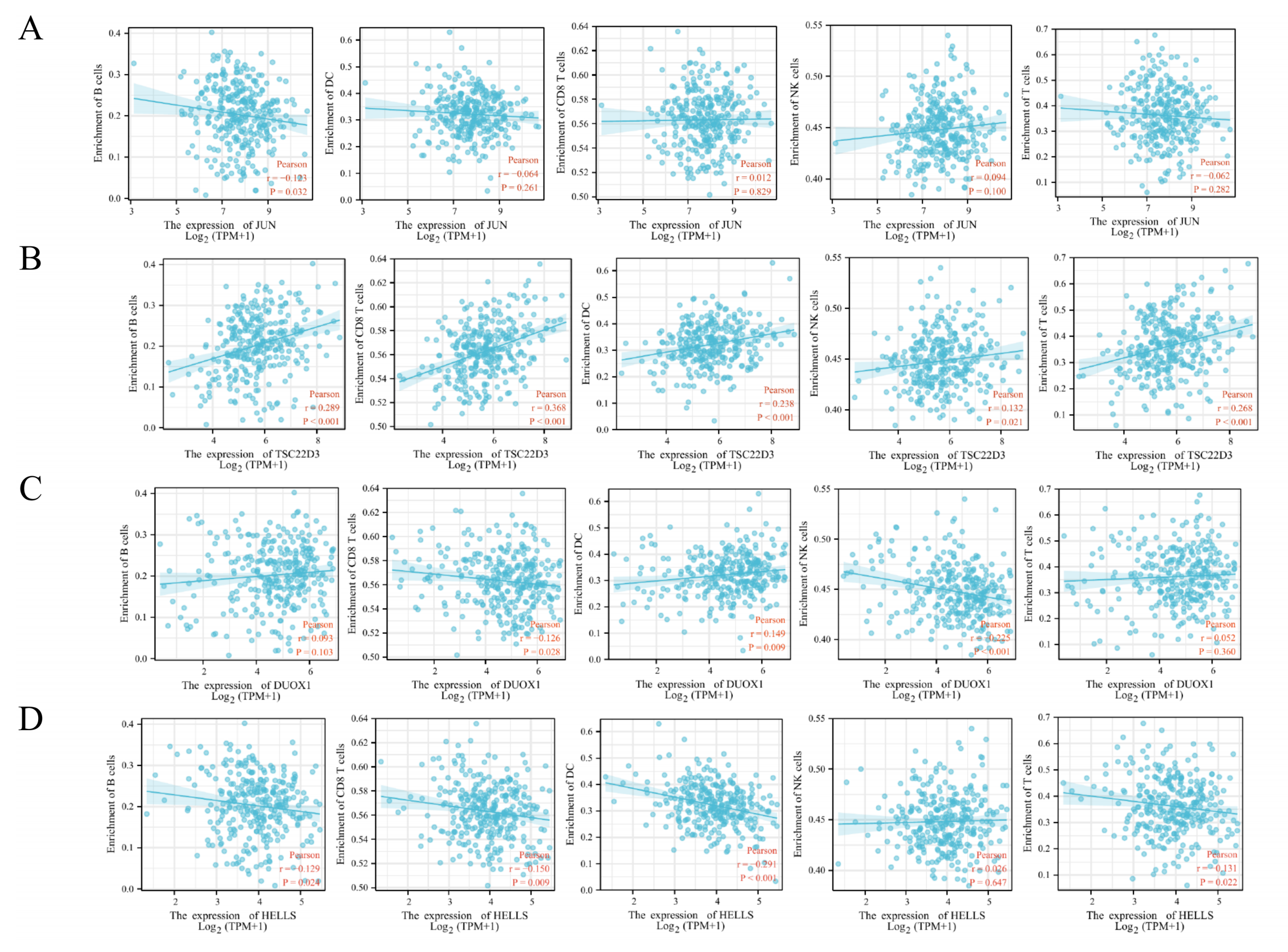

Supplement: Supplementary file 4 [file Image6.TIF]

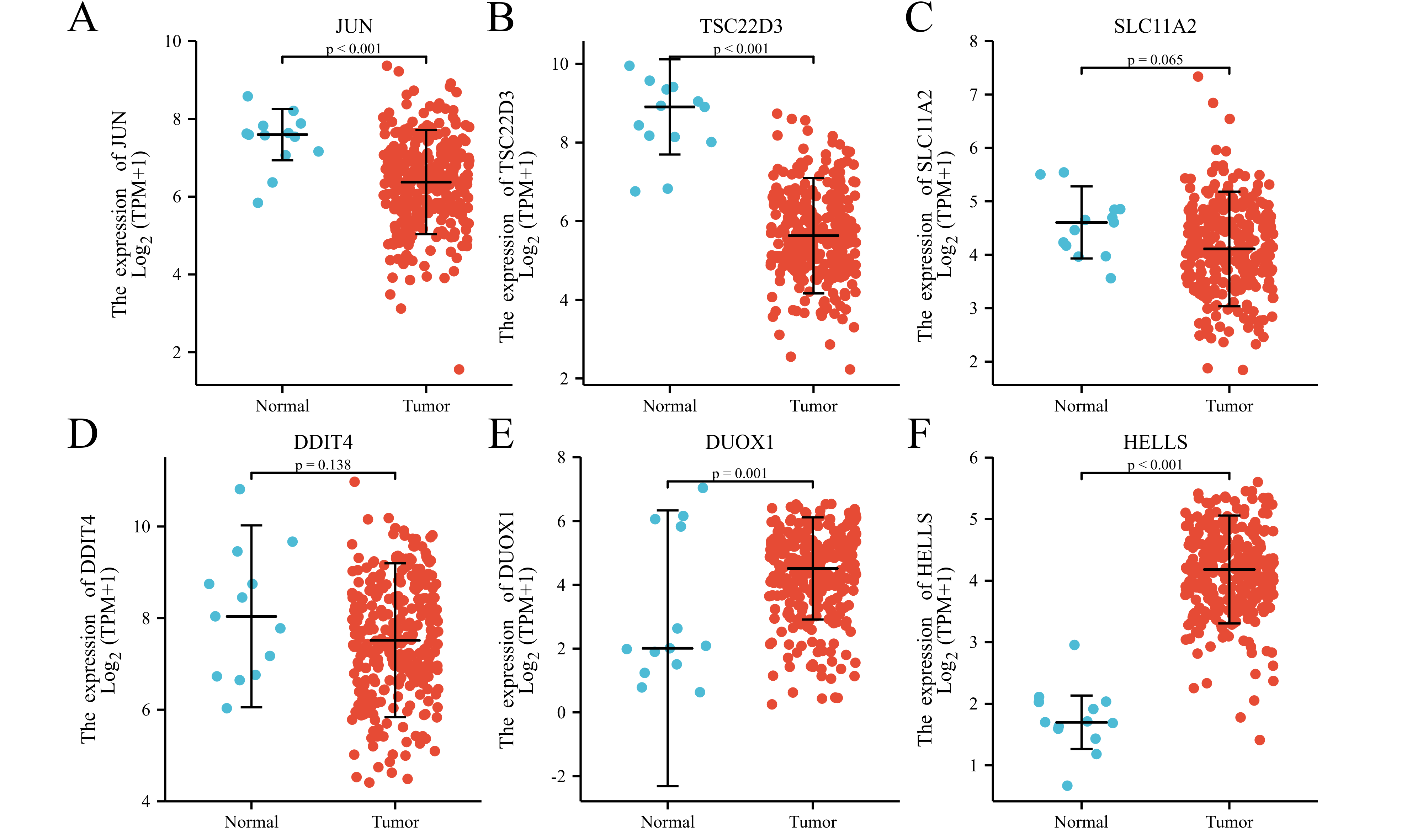

Supplement: Supplementary file 5 [file Image5.TIFF]

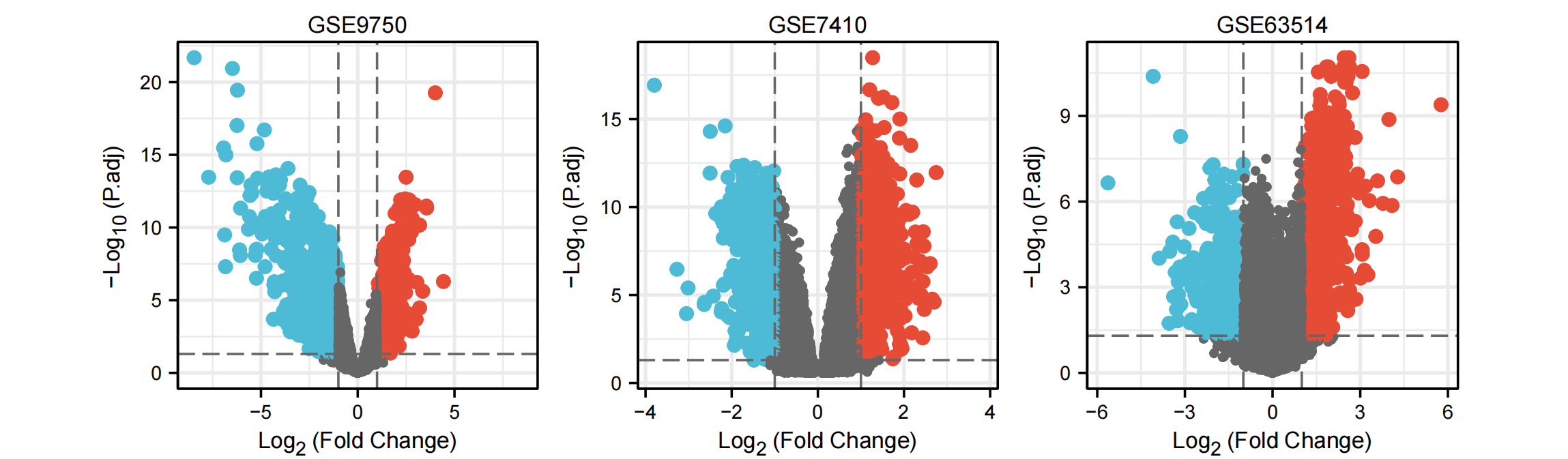

Supplement: Supplementary file 6 [file Image1.TIF]

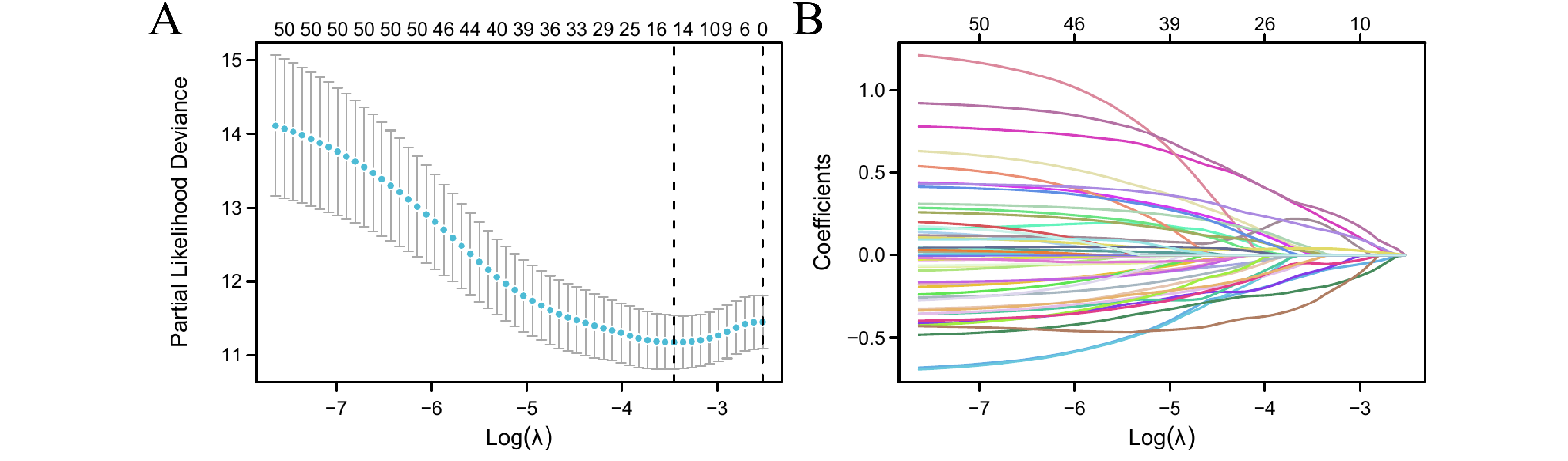

Supplement: Supplementary file 11 [file Image2.TIFF]

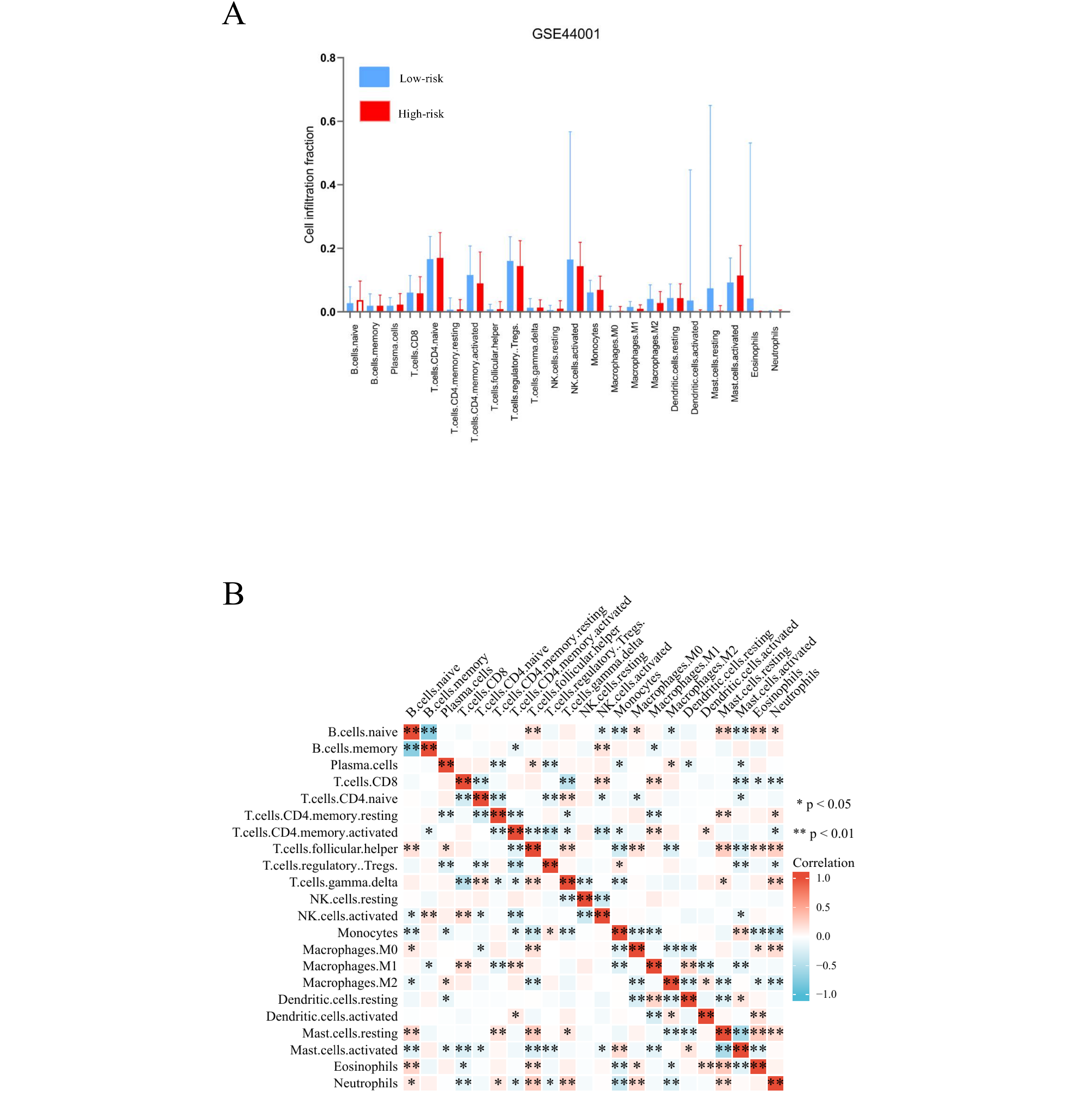

Supplement: Supplementary file 12 [file Image4.TIFF]
